# The "No-touch" Harvesting Technique Revives the Position of the Saphenous Vein as an Important Conduit in CABG Surgery: 30-year Anniversary

**DOI:** 10.21470/1678-9741-2021-0959

**Published:** 2021

**Authors:** Bruno Botelho Pinheiro, Michael Dashwood, Domingos S. R. Souza

**Affiliations:** 1 Department of Cardiovascular Surgery, Hospital do Coração Anis Rassi, Goiânia, Goiás, Brazil.; 2 Surgical and Interventional Sciences, Royal Free Hospital Campus, University College London Medical School, London, United Kingdom.; 3 Department of Cardiothoracic and Vascular Surgery, Faculty of Medicine and Health, Örebro University, Örebro, Sweden.

For many reasons, the saphenous vein (SV) and internal thoracic artery (ITA) will continue to be the most important conduits in coronary artery bypass grafting (CABG). The high incidence of vein graft occlusion is an important and unsolved problem in myocardial revascularization^[1]^. Improvement of SV graft patency rate is therefore a great challenge in the field of cardiovascular surgery. The widespread use of complete arterial grafting with the left internal thoracic artery and other arterial conduits cannot yet be justified on scientific grounds, as there are few late survival data and controlled studies to support this statement^[2]^.

Different structural and functional properties between arteries and veins may explain the better results of the arterial grafts. Nevertheless, the trauma to the SV wall that occurs during its harvesting for CABG may also contribute to the poor results of venous grafts^[3]^. At time of implantation, adventitial and endothelial damage is almost absent in ITA grafts. In contrast, the endothelium of conventionally-harvested SV shows greater thrombogenic properties and the SV adventitial layer is often completely removed during the operation^[4,5]^. Usually, the vein is harvested traumatically by a junior of the surgical team, with least experience, a situation likely to contribute to the poor quality of the graft and consequently to graft failure.

Many strategies have been introduced in an attempt to prevent vein graft occlusion and to improve short- and long-term patency rate. Apart from established adjuvant medical therapy, new pharmacological agents, gene therapy, as well as mechanical devices are presently undergoing evaluation. Many of these strategies have shown promise in experimental vein bypass models. However, few have successfully been translated into clinical practice. We believe that these approaches represent the familiar scenario of closing the stable door after the horse has bolted. Surely, it is more desirable to prevent damage to the vein in the first place than to correct the damage once it has been inflicted. This damage leads to neointimal hyperplasia and other aspects of graft failure. There is a wealth of literature showing the adverse effects of surgical trauma, especially the harmful effects of high pressure distension that is often used during vein graft harvesting and the subsequent fate of the vein graft^[4]^.

The no-touch (NT) technique, where the SV is harvested with its pedicle of surrounding tissue intact, was developed in the early 1990s by Domingos Souza in the department of Anesthesiology and Cardiothoracic Surgery at Örebro University Hospital, Sweden^[6]^. The main aim, when applying NT harvesting, is to maintain the structural and functional integrity of the vein wall and consequently to improve SV graft outcome. After a few pilot cases, a randomized single-center clinical trial was initiated in 1993 to compare conventional saphenous vein grafts with no-touch saphenous vein grafts (NTSVG). In this trial, we followed the patients over three different time points; at mean time 1.5, 8.5, and 16 years after the operations^[7]^. The results of this trial showed favourable for the NT technique regarding both angiographic^[7]^ and clinical outcomes^[8]^. Parallel to the clinical trials many basic research studies were performed in the Dashwood laboratory at the Royal Free Hospital/University College London, United Kingdom. These studies have revealed certain mechanisms underlying the improved performance of NTSVGs, in particular the protective role of the perivascular fat (PVF) that preserves both structural integrity and functional properties of the vein wall^[9-11]^.

Based on our results, it was concluded that the method of harvesting greatly influences the fate of the SV graft. The PVF and adventitial layer contain structures possessing both mechanical and functional properties that protect the vein from spasm and ischemia^[4,5,10]^. Furthermore, endothelial integrity was better maintained using the NTSVG. Nitric oxide synthase activity was preserved on the endothelium and was derived from both neuronal and adventitial microvessel sources, suggesting that nitric oxide (NO) availability is retained by these grafts. The vasorelaxant and thromboresistant activities of NO may be responsible for the reduced spasm and the observed improvement of the patency rate of NTSVGs^[9,10]^. In addition, the surrounding tissue possesses mechanical properties that supports excessively long vein grafts and prevents kinking^[5]^.

Initially there appeared little acceptance for this technique with it being used solely in the department of thoracic and cardiac surgery at Örebro University Hospital, where it was developed. Following publication of both the randomized clinical trials and the basic science studies, considerable interest has been raised worldwide whereby the NT technique is recognized and has been adopted in many countries, especially in Sweden^[12,13]^^),^ Japan^[14]^, China^[15]^, and South Korea^[16]^. Currently, two large randomized multicenter trials are ongoing to evaluate the NT technique. The first one is being performed in China, which included 2,655 patients and the one-year results was recently published^[15]^. The second similar ongoing randomized trial is in Sweden and 900 patients were included^[13]^. However, the data of this trial will not be analyzed until 2023. The results of these studies are expected to be of great value for the further spread and adoption of the NT technique particularly because any improvement in SV graft performance needs to be easily applied as well as provides similar good results in the majority of cardiac centers worldwide.

Following the first publication describing the NT technique, this technique has been the subject of four theses and many publications from around the world. Furthermore, NTSVG has been referred to in many articles published in peer-reviewed journals. Some years passed before the advantages of the NTSVG technique was recognized, and today it is ranked as class IIa in the European Society of Cardiology/European Association for Cardio-Thoracic Surgery Guidelines on myocardial revascularization^[17]^.

Concerns have been raised regarding the issue of increased leg-wound infection. The NT technique consists not only of harvesting the vein with a fat pedicle intact but also of other important steps, one of which is to preoperatively mark the course of the vein on the overlying skin by using an ultrasonographic doppler assessment^[18]^. This allows us to make the skin incision exactly above the vein, thereby reducing soft tissue injury and the creation of flap tissues. In addition, this procedure allows selection of the best vein segments suitable for grafting.

By implementing the technique as described previously^[18]^, we have managed to reduce wound healing complications considerably. It would certainly be preferable to obtain a conduit with a longer patency, even if there is a higher risk of wound infection^[19]^. The ideal situation would be to obtain a superior conduit combined with minimal risk of harvesting site infection. Therefore, development of a minimally invasive endoscopic technique for harvesting the NT veins is essential in order for the NT technique to be more widely accepted. Fortunately, a number of recent publications have addressed this important issue whereby the SV is removed with minimal vascular damage, with PVF intact, and reduced wound complications^[20]^.

After all scientific evidence during the last 30 years, the NT SV should be recognized as a very important conduit for CABG surgery.
